# Genotype Complements the Phenotype: Identification of the Pathogenicity of an LMNA Splice Variant by Nanopore Long-Read Sequencing in a Large DCM Family

**DOI:** 10.3390/ijms232012230

**Published:** 2022-10-13

**Authors:** Farbod Sedaghat-Hamedani, Sabine Rebs, Elham Kayvanpour, Chenchen Zhu, Ali Amr, Marion Müller, Jan Haas, Jingyan Wu, Lars M. Steinmetz, Philipp Ehlermann, Katrin Streckfuss-Bömeke, Norbert Frey, Benjamin Meder

**Affiliations:** 1 Institute for Cardiomyopathies Heidelberg (ICH), University Hospital Heidelberg, 69120 Heidelberg, Germany; 2 DZHK (German Centre for Cardiovascular Research), Partner Site Heidelberg and Mannheim, 69120 Heidelberg, Germany; 3 Department of Internal Medicine III, University Hospital Heidelberg, 69120 Heidelberg, Germany; 4 Department of Cardiology and Pneumology, Georg-August-University Göttingen, 37073 Göttingen, Germany; 5 DZHK (German Centre for Cardiovascular Research), Partner Site Göttingen, 37075 Göttingen, Germany; 6 Department of Pharmacology and Toxicology, University of Würzburg, 97070 Würzburg, Germany; 7 Department of Genetics, Stanford University, Stanford, CA 94305, USA; 8 Clinic for General and Interventional Cardiology/Angiology, Herz- und Diabeteszentrum NRW, University Hospital of the Ruhr-Universität Bochum, 32545 Bad Oeynhausen, Germany

**Keywords:** familial DCM, laminopathy, long-read sequencing, nanopore, induced pluripotent stem cell cardiomyocytes

## Abstract

Dilated cardiomyopathy (DCM) is a common cause of heart failure (HF) and is of familial origin in 20–40% of cases. Genetic testing by next-generation sequencing (NGS) has yielded a definite diagnosis in many cases; however, some remain elusive. In this study, we used a combination of NGS, human-induced pluripotent-stem-cell-derived cardiomyocytes (iPSC-CMs) and nanopore long-read sequencing to identify the causal variant in a multi-generational pedigree of DCM. A four-generation family with familial DCM was investigated. Next-generation sequencing (NGS) was performed on 22 family members. Skin biopsies from two affected family members were used to generate iPSCs, which were then differentiated into iPSC-CMs. Short-read RNA sequencing was used for the evaluation of the target gene expression, and long-read RNA nanopore sequencing was used to evaluate the relevance of the splice variants. The pedigree suggested a highly penetrant, autosomal dominant mode of inheritance. The phenotype of the family was suggestive of laminopathy, but previous genetic testing using both Sanger and panel sequencing only yielded conflicting evidence for *LMNA* p.R644C (rs142000963), which was not fully segregated. By re-sequencing four additional affected family members, further non-coding *LMNA* variants could be detected: rs149339264, rs199686967, rs201379016, and rs794728589. To explore the roles of these variants, iPSC-CMs were generated. RNA sequencing showed the *LMNA* expression levels to be significantly lower in the iPSC-CMs of the *LMNA* variant carriers. We demonstrated a dysregulated sarcomeric structure and altered calcium homeostasis in the iPSC-CMs of the *LMNA* variant carriers. Using targeted nanopore long-read sequencing, we revealed the biological significance of the variant c.356+1G>A, which generates a novel 5′ splice site in exon 1 of the cardiac isomer of *LMNA*, causing a nonsense mRNA product with almost complete RNA decay and haploinsufficiency. Using novel molecular analysis and nanopore technology, we demonstrated the pathogenesis of the rs794728589 (c.356+1G>A) splice variant in *LMNA*. This study highlights the importance of precise diagnostics in the clinical management and workup of cardiomyopathies.

## 1. Introduction

Dilated cardiomyopathy (DCM) is one of the leading causes of heart failure, sudden cardiac death (SCD) and arrhythmia [1]. It is defined as a disease of the heart muscle, characterized by left ventricular or biventricular dilation with systolic dysfunction in the absence of either volume or pressure overload or coronary artery disease that is sufficient enough to explain the dysfunction [2]. However, the clinical phenotypes, morbidity, disease progression, and outcomes vary considerably depending on the etiology of the disease. Identifying the correct etiology can be crucial for the clinical management of patients and their family members. Moreover, it improves patients’ clinical outcomes by enabling the deployment of a personalized treatment approach [3].

Secondary DCM due to myocarditis, for instance, shows a better outcome compared to DCM patients with pathogenic *LMNA* variants, with SCD, arrhythmia and heart transplantation more often being endpoints in the case of laminopathies [4]. Approx. 30% of DCM cases are familial, and more than 50 different genes are linked to DCM in experimental models or humans [1,4]. In the last decade, next-generation sequencing (NGS) has led to increased detection rates of pathogenic variants [1]. However, this has also increased the number of variants with unknown significance (VUS). Many DCM variants have not been functionally analyzed. Thus, the precise pathomechanisms of the variants in terms of the disease susceptibility and phenotype expression, including heart failure (HF) development or SCD, remain unknown [5]. 

In the present study, we deployed an innovative diagnostic approach to identify a pathogenic *LMNA* variant in a large DCM family that was undiscovered for many years using conventional diagnostic methods. The results of this study highlight the potential benefits of utilizing nanopore long-read sequencing technology and patient-specific induced-pluripotent-stem-cell-derived cardiomyocytes (iPSC-CMs) in future diagnostic workups of cardiomyopathy patients.

## 2. Results

### 2.1. Patients’ Phenotypes Were Suggestive of a Laminopathy

**Figure 1:**
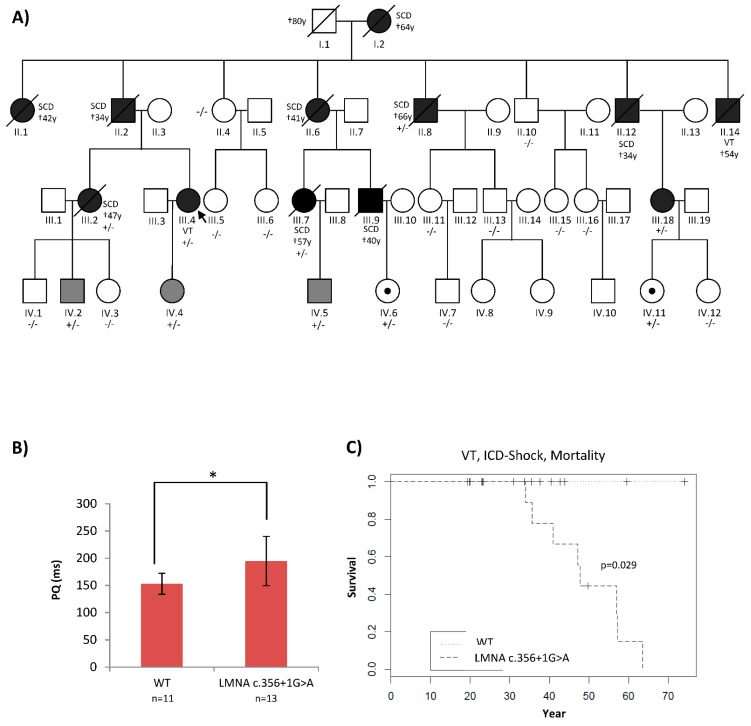
(**A**): **Patient’s pedigree showing co-segregation of an *LMNA* splice site mutation, *LMNA* c.356+1G>A**. The index patient was diagnosed with DCM and conduction disease of 40 years and underwent ICD implantation. At age 47, he received an adequate ICD therapy. +/− is heterozygous, −/− is the homozygous wild type. Black: full phenotype, grey: mild phenotype, dot: genotype positive/phenotype negative. Symbol with a diagonal line: Deceased. Dagger indicates the age of death. (**B**,**C**): Patients who carried *LMNA* c.356+1G>A had significantly longer PQ intervals and higher rates of ventricular arrythmia and mortality. * *p* < 0.05.

A four-generation family underwent deep phenotyping at our Institute for Cardiomyopathy Heidelberg (ICH). The inheritance pattern suggested an autosomal dominant and highly penetrant disease variant. The etiology of the disease had remained unclear since the first medical encounter in 1970s. Sudden cardiac death occurred in nine family members between the ages 34 and 66 years (Figure 1A and Table 1). No neuromuscular disorders were observed, but conduction disease, as well as arrhythmia, were typical hallmarks of their cardiac phenotype (Figure 1B,C).

### 2.2. Traditional Diagnostic Methods Revealed Two LMNA Variants with Unclear Pathogenicity

Although the phenotype of this family hinted at a laminopathy, gene testing could not identify a clear pathogenic variant. Using Sanger sequencing and Haloplex NGS, R644C (rs142000963) in LMNA could be identified in seven family members. However, this variant showed incomplete and conflicting co-segregation with the DCM phenotype (Table 1). Through the HaloPlex NGS of all 84 cardiomyopathy-related genes, another rare LMNA variant was detected (rs149339264: c. C1312T). This rare variant was predicted to be a splice acceptor in the case of one transcript isomer (ENST00000361308) and was predicted to cause the aberrant splicing of exons 7 and 8. As a result, it arrived at a premature stop codon at the novel transition from exon 7 and 8. In the case of the other LMNA isomers, this variant was predicted to be synonymous. Surprisingly, this variant (rs149339264) could not be validated by Sanger sequencing, showing only the wild-type sequence. Investigating this issue in more detail confirmed that this was due to allelic dropout because of two intronic variants that interfere with the sequencing primer (SNP–primer mismatch) (Supplementary Figure S1A). The allele containing the rare variant could not be amplified, so that only the wild-type allele was amplified and sequenced.

### 2.3. High-Coverage NGS (SureSelect) Detected Four LMNA Variants

After the re-evaluation of this family at our institute and the re-sequencing of all family members by high-coverage NGS (SureSelect), two additional rare intronic (rs199686967 and rs201379016) variants and one potential splice variant (LMNA c.356+1G>A, rs794728589) on the same allele of LMNA were found that fully co-segregated in the family. The reason why the splice variant rs794728589 could not be found in the HaloPlex assay was that the wrong annotation of the exon/intron boundary was identified in the HaloPlex assay (Supplementary Figure S1B). As the roles of these LMNA variants in the disease mechanism were still unknown, and the various conflicting results were lowering our confidence in the results to be reported to the family, patient-specific iPSC-cardiomyocytes (iPSC-CMs) were generated from two affected family members (III.18 and IV.2) (Figure 1A) for further investigations.

### 2.4. Functional Analyses Revealed the Pathogenicity of rs794728589

**Figure 2:**
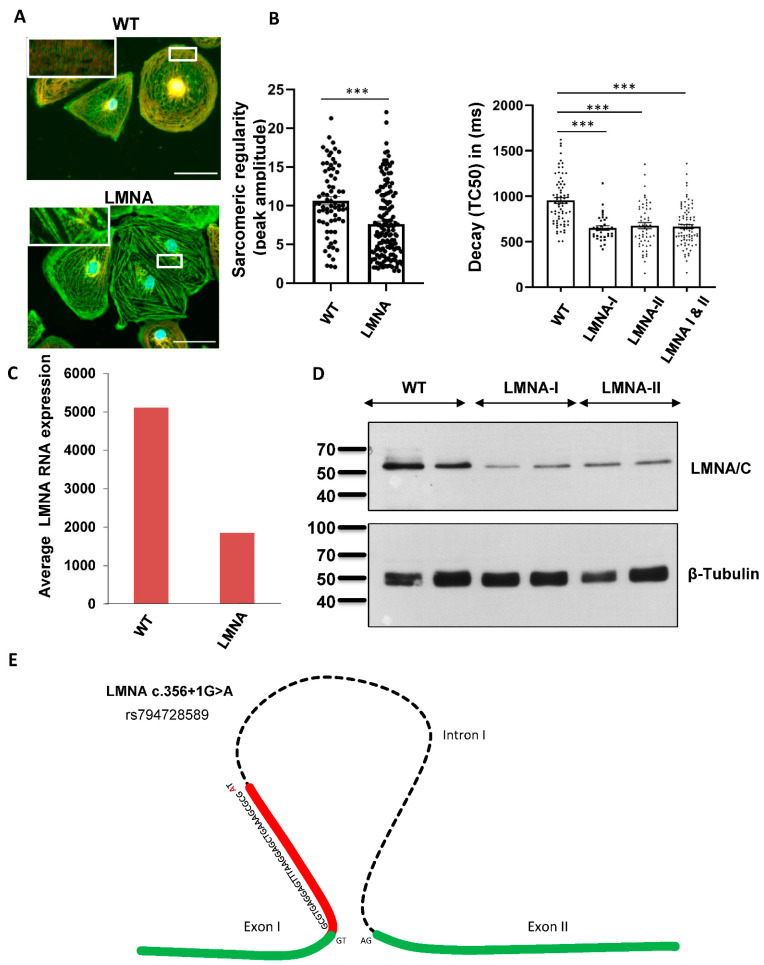
**Identifying a novel LMNA variant using precise diagnostic methods. (A) Disturbed sarcomeric z-disc regularity in iPSC-CMs with the LMNA variant**. The sarcomeric structure was visualized by immunofluorescent staining with α-actinin (green) and TTN-M8/M9 (red). Scale bar = 50 µm (white box). Using fast Fourier transformation (FFT), the sarcomeric pattern regularity of the α-actinin channel was analyzed. Four cardiac differentiations for the control (*n* = 72) and 8 differentiations for 2 patient iPSC-LMNA lines (*n* = 145) were analyzed. Data are presented as mean ± SEM, *p* < 0.001 according to Student’s *t*-test. (**B**) Ca^2+^ transient decay time is decreased in LMNA-iPSC-CMs. Ca^2+^ cycling was assessed using Ca^2+^-sensitive probe Fluo4. Values for the transient decay time are shown with 4 cardiac differentiations of for the control (*n* = 70), 2 differentiations for LMNA 1 (*n* = 35) and 3 differentiations for LMNA 2 (*n* = 53). *** *p* < 0.001. (**C**) mRNA decay of LMNA. LMNA expression was significantly downregulated in iPSC-CMs with the LMNA c.356+1G>A splice mutation. By Western blot analysis of iPSC-CMs, the downregulation of LMNA in patients with the splice mutation (LMNA c.356+1G>A) could be validated (**D**). (**E**) Schematic illustration of LMNA c.356+1G>A mutation. The G to A transversion induces the activation of a cryptic donor splice site in exon 1 and causes a 32 bp-long frameshift deletion, which leads to a premature stop.

iPSC-CMs were generated from two affected family members (III.18 and IV.2) (Supplemental Figure S2). Double staining was used to visualize the sarcomeric Z-disc with α-actinin and the M-line with TTN M8/M9 antibodies, followed by the quantification of the sarcomeric regularity using fast Fourier transformation (FFT). We observed a significant impairment in the sarcomeric regularity for the Z-disc in the LMNA-CMs compared to the control CMs (Figure 2A). To analyze the calcium homeostasis, we used the cytosolic Ca^2+^-sensitive dye Fluo-4 and observed decreased decay times for the LMNA-iPSC-CMs compared to the control cells under basal conditions (Figure 2B), whereas the rise time did not change in the LMNA-iPSC-CMs (Supplementary Figure S3A). Additionally, the iPSC-CMs were stimulated with Isoprenaline (Iso) to test the β-adrenergic stimulation capacity of the generated CMs. The control and LMNA-CMs demonstrated a prominent decrease in the rise and decay times after Iso treatment (Supplementary Figure S3B,C). Based on the RNA sequencing of these iPSC-CMs and wild-type controls, the LMNA expression levels were shown to be significantly lower in the LMNA variant carriers (Figure 2C,D). Targeted nanopore long-read sequencing underlined the biological significance of the variant c.356+1G>A (rs794728589), which generates a novel 5′ splice site in exon 1 of the cardiac isomer of LMNA, causing a nonsense mRNA product with almost complete RNA decay, resulting in haploinsufficiency in LMNA (Figure 2E).

## 3. Discussion

Each individual is genetically and epigenetically unique. Precision medicine for DCM, by implementing multi-omics approaches, helps us to better understand the disease mechanism and improve clinical decision making and the risk stratification of affected index cases and family members [3]. Familial forms of DCM often remain undiagnosed, especially due to a lack of detailed familial history or pedigree information. Adequate family screening and precise genetic evaluation are necessary in order to adequately inform the family and guide decision making [6]. Using a proactive strategy helps to identify DCM patients at an earlier stage and improve their survival, especially those with mutations in high-risk genes such as LMNA, PLN, SCN5A or RBM20 [4,7,8].

Patients with LMNA mutations present with a progressive disease characterized by conduction abnormalities and fatal arrhythmias [9]. In this family, before the identification of the LMNA variants, nine family members experienced SCD, while seven of them had no preceding signs of heart failure or life-threatening arrhythmia. However, five of these nine patients had received a pacemaker prior to the incident due to a diagnosis of conduction disease. Once LMNA mutation carriers develop clear DCM phenotypes, the course of the disease becomes more malignant, presenting with a higher incidence of lethal arrhythmia [4]. Thus, in patients with LMNA mutations who are eligible for pacemaker implantation due to their severe conduction disease, ICD implantation is recommended in order to prevent SCD following lethal arrhythmia [6,10]. This highlights the importance of genetic testing for patients presenting with a history of familial SCD or malignant arrhythmia, particularly in the presence of prior supraventricular tachycardia (SVT) or conduction abnormalities. Even minimal ECG abnormalities, such as first-degree atrioventricular blocks or left fascicular blocks, in families with DCM and SCD are indicative of a disease-causing LMNA variant.

In this study, we identified and validated a previously undetected splice LMNA variant (c.356+1G>A) in a large DCM family. The genetic etiology of the disease had remained undetected for nearly four decades because of the allelic dropout in Sanger sequencing and low coverage in previous versions of NGS (HaloPlex). Allelic dropout is one of the major reasons for false negative genetic results in individuals, caused by a reduction in the efficiency of PCR-based targeted sequencing [11]. In fact, 2 to 5% of the long-QT syndrome patients have a negative genetic finding due to this phenomenon [12]. This point suggests that the genetic tests previously performed using conventional techniques that yielded negative results in evident cases of cardiomyopathies do not rule out a genetic cause of the disease, especially in familial cases. Re-sequencing using advanced techniques should be considered in such cases [11]. Regular, updated research on the single-nucleotide variant distribution and improvements in the primer design algorithms should also be implemented routinely in order to improve the diagnostic accuracy of genetic testing [11]. 

Over the last few years, nanopore technology has impacted the field of genetic sequencing and has become an increasingly important addition to the family of third-generation sequencing technologies [13]. Generating long reads, real-time sequencing and fast library preparation are major advantages of this sequencing technique [14]. Bowden et al. showed, for the first time, the accuracy of the genotyping of human samples by nanopore sequencing [15]. Leija-Salazar et al. also detected missense variants and intronic deletions in the GBA gene for Gaucher disease [16]. However, ongoing improvements on variant-calling and read mapping techniques are essential for the use of nanopore technology for clinical purposes [15]. Numerous diseases, including cardiovascular diseases, are associated with alternative splicing dysregulation. While the conventional short-read RNA sequencing method can only detect expression changes in the whole gene or individual exons [17], nanopore technology provides a novel tool for the more comprehensive analysis of alternative splicing. Zhu et al. recently identified, using full-length transcript isoform and nanopore sequencing, 121 differentially expressed transcript isoforms in 107 cardiac genes in patients with DCM and *RBM20* pathogenic variants [17]. The current study serves as an example of the potential benefits of using nanopore technology in genetic testing for the identification of disease mechanisms in challenging cases. 

By applying novel, precise diagnostic methods, including iPSC-CMs and nanopore RNA sequencing, we identified and evaluated the pathomechanism of the disease in this large family and identified the LMNA c.356+1G>A splice variant as a disease-causing variant. These results are of great importance for the accurate treatment of these patients and the ability to confirm or rule out the diagnosis in other family members. With future clinical and preclinical therapies for laminopathies, such as p38 inhibitors and gene editing, these patients could benefit by receiving personalized therapies instead of/along with conventional heart failure therapy [18,19]. 

## 4. Material and Methods

### 4.1. DNA and RNA Sequencing

The isolation of DNA from blood was performed using DNA Blood Maxi, DNeasy Blood and Tissue (Qiagen, Venlo, Netherlands). The isolated DNA was then quantified using the Qubit^®^ dsDNA HS Assay with a Qubit fluorometer (ThermoFisher, Waltham, MA, U.S.). In total, 150 ng of high-molecular DNA was used to generate sequencing libraries for Illumina sequencing using Agilents SureSelect Target Enrichment technology. Sequencing was performed using a HiSeq2000 or NovaSeq 6000 (paired end 2 × 100 bp). For the isolation of RNA, AllPrep^®^ DNA/RNA/Protein Mini (Qiagen) was used. The quantification and quality assessment were performed using the Fragment Analyzer HS-RNA Kit with a Fragment Analyzer (Agilent, Santa Clara, CA, U.S.). RNA sequencing libraries were generated using TruSeq Stranded Total RNA Library Präp (Illumina, California, U.S.). Sequencing was performed using a HiSeq2000 (paired end 2 × 75 bp). 

The pellets of iPSC-CMs were snap-frozen in liquid nitrogen and stored at −80 °C for DNA and RNA isolation. Genomic DNA was isolated using the QIAMP DNA Kit (Qiagen) and RNA was isolated using the SV Total RNA-Isolation Kit (Promega, Wisconsin, United States), according to the manuals’ instructions. To remove residues of genomic DNA after RNA isolation, the samples were incubated with 2 U TURBO DNAse (Thermo) for 30 min at 37 °C.

### 4.2. Long-Read RNA Sequencing of cDNA Libraries 

The DNase treatment of the RNA was performed using ZymoResearch RNA Clean and Concentrator-5. Purified RNAs (5 ng) were mixed with 1 µL oligodT (10 µM, /5SpC3/ A*A*G*CAGTGGTATCAACGCAGAGTACTTTTTTTTTTTTTTTTTTTTTTTTTTTTT), 1 µL dNTP (10 mM) and 0.1 µL of Takara recombinant ribonuclease inhibitor (40 U/µL) in a total volume of 5 µL and were subsequently incubated at 72 °C for 3 min, 4 °C for 10 min and 25 °C for 1 min. Another 5.5 µL mixture containing 2 µL of 5× SuperScript II buffer, 2 µL of 5 M betaine, 0.5 µL of 100 mM DTT, 0.5 µL of SuperScript II (200 U/µL), 0.25 µL of Takara recombinant ribonuclease inhibitor, 0.1 µL of 100 µM TSO, 0.09 µL of nuclease-free water and 0.06 µL of 1 M MgCl_2_ were added. The total 10 µL of RT reaction mixture was incubated at 42 °C for 90 min, with 10 cycles of 50 °C for 2 min and 42 °C for 2 min. The reaction was then stopped by incubation at 70 °C for 15 min and held at 4 °C. The solution was then treated with 1 µL of 1:10 dilution of NEB RecJf (Catalog # M0264S) at 37 °C for 30 min and 65 °C for 20 min.

### 4.3. Generation of Patient-Specific Induced Pluripotent Stem Cells (ps-iPSCs)

Skin fibroblasts were isolated from skin biopsies, as described earlier [20]. For the plasmid-based integration-free reprogramming, the protocol described earlier was used [20]. Briefly, 5 × 10^5^ cells were subjected to electroporation using the NHDF Nucleofector Kit (Lonza). Cells were suspended in nucleofection solution, and 1µg samples of the plasmids pCXLE-hSK, pCXLE-hUL and pCXLEhOct3/4-shp53-F were used per experiment. These factors were encoded on three episomal plasmid vectors, as published by the Yamanaka group [21]. The protocol for Sendai-based transduction was described earlier [20]. Briefly, 3 × 10^5^ fibroblasts were seeded in 2 wells of a 6-well or 12-well plate one day before transduction. Sendai virus from the CytoTune™-iPS 2.0 Sendai Reprogramming Kit (Thermo Fisher Scientific, Waltham, MA, USA) was used at an MOI of 5/5/3 (KOS/hc-myc/hKLF4) and added to the cells in fresh medium. For every patient, two iPSC lines were selected and cultured for the subsequent differentiation into iPSC-CMs. 

### 4.4. Cell Culture and Cardiac Differentiation of iPSCs

The iPSC lines were cultured in Essential 8 medium (Thermo) at 37 °C in a 5% CO_2_ atmosphere. The cardiac differentiation of the iPSCs was performed by sequential targeting of the WNT pathway, as described earlier [20]. Briefly, the iPSCs were cultured in 12-well plates as a monolayer until they reached the confluency of 80–90% required to initiate the differentiation. For this, the medium was changed to differentiation medium composed of RPMI 1640-GlutaMAX (Thermo) supplemented with 0.02% ascorbic acid and 0.05% albumin (Sigma-Aldrich, St. Louis, MO, U.S.), which also included CHIR99021 (4 µmol/L, Merck Millipore, Burlington, MA, U.S.) (day 0). After 48 h, the medium was replaced with differentiation medium containing 5 µmol/L inhibitor of Wnt-production (IWP2, Millipore) (day 2) for 2 days. On day 10, the medium was exchanged for cardio culture medium composed of RPMI 1640 (Thermo) supplemented with B27 and insulin (Life Technologies, California, U.S.). On day 20, the iPSC-CMs were purified by metabolic selection for 2 to 4 days using 4 mmol/L lactate as a carbon source. All experimental studies were performed on the iPSC-CMs at days 60–90 after the start of the differentiation.

### 4.5. Assessment of Sarcomeric Regularity of iPSC-CMs

Cultures at different stages were fixed with 4% paraformaldehyde (PFA, Sigma) at room temperature for 20 min and then blocked with 1% BSA in PBS at 4 °C overnight. Immunofluorescent staining of α-Actinin (1:1000, Sigma) and Titin-M8/M9 (1:750, MyoMedix) were obtained using a 40× objective. Subsequently, the α-Actinin channel was analyzed through fast Fourier transformation (FFT) in ImageJ for the striation pattern regularity. The bar graphs depict the height of the first-order peak amplitude at the spatial frequency.

### 4.6. Calcium Imaging: Fluo-4

For the analysis of the Ca^2+^ kinetics, cytosolic calcium (Ca^2+^) transients were imaged using Fluo-4 probe (Invitrogen). For this purpose, 300.000 iPSC-CMs were plated onto Geltrex-coated 25 mm round coverslips. After 7–9 days, the iPSC-CMs were incubated with 2.5 µM of Ca^2+^-sensitive fluorescent probe Fluo-4 for 30 min in Tyrode’s solution containing 140 mM NaCl, 5.4 mM KCl, 1 mM MgCl2, 10 mM glucose, 1.8 mM CaCl_2_ and 10 mM HEPES (pH 7.4). The iPSC-CMs were paced at 0.25 Hz, and Ca^2+^ transients were recorded at room temperature using a Zeiss LSM Axio Observer Z1 confocal microscope with the following settings: a laser of 488 nm with 0.4–1.8%, a line scan mode with 20,000 cycles and zoom factor of 3. 

### 4.7. Protein Analysis

Proteins were extracted from the iPSC-CMs and prepared for SDS-PAGE, followed by specific immunoblotting. In detail, protein samples were separated using hand-cast polyacrylamide gels (12% separating gel, 5% stacking gel, Mini-PROTEAN^®^ Cell Biorad, California, U.S.) in 1× running buffer (in mM): 25 Tris, 192 glycine, 0.1% SDS. The separated proteins were transferred to a PVDF membrane in 1× blotting buffer (in mM): 19 Tris, 144 glycine, pH 8.3, 15% methanol. To avoid unspecific binding, the membranes were blocked with 5% BSA in TBST buffer. Immunostaining was performed with a polyclonal antibody against Lamin A/C (1:200, Santa Cruz #sc-20681) followed by secondary HRP antibody (1:3000, Cell Signaling #7074). After staining, chemiluminescent substrate (ECL Western Blotting Substrate Pierce™ #32106) was used to detect the HRP activity of the antibodies. 

## 5. Conclusions

This study shows exemplifies how deep phenotyping, in combination with innovative molecular techniques, can be of clinical benefit. By generating iPSC-CMs and performing long-read nanopore sequencing, a diagnosis can be reliably established. The findings have significant clinical relevance to living family members, as genotype-positive family members will receive a better SCD risk stratification, and an ICD device therapy will be reconsidered after combining the genotype information with the clinical risk features. 

## Figures and Tables

**Table 1:** Clinical characteristics of the family members.

ID	Age	Gender	Affected	Splice Mutation	R644C Variant	Age at Onset	DCM	NYHA	EF	Rhythm	Conduction Disease	Outcome
**I.2**	64	F	Yes	(+/−)	NA	57	NA	NA	NA	AF	AVB-III°	PM 60y, SCD 64y
**II.1**	42	F	Yes	NA	NA	37	NA	NA	NA	AF	-	PM 40y, SCD 42y
**II.3**	34	M	Yes	(+/−)	NA	32	NA	NA	NA	AF	-	SCD 34y
**II.4**	72	F	No	−/−	+/−	-	No	I	60	AF	-	-
**II.6**	41	F	Yes	(+/−)	NA	37	NA	NA	NA	AF	AVB-III°	PM 37y, SCD41y
**II.8**	66	M	Yes	+/−	+/−	55	Yes	III	35	AF	AVB-I°, LAH	PM 59y, SCD 66y
**II.10**	77	M	No	−/−	+/−	-	No	II	58	SR	-	-
**II.12**	66	M	Yes	(+/−)	NA	28	NA	NA	NA	SR	AVB-II°	PM 30y, SCD 34y
**II.14**	54	M	Yes	NA	NA	36	NA	NA	NA	NA	AVB-III°	PM 51y, VT, ICD 52y, Death 54y
**III.2**	45	F	Yes	+/−	-	44	Yes	I	40	SR	AVB-I°, LAH	nsVT, ICD 45y, SCD 47y
**III.4**	52	F	Yes	+/−	-	40	Yes	III	50	AF	AVB-I°, LBBB	Prophylactic ICD 40y, VT and ICD therapy 47y, CRT-D 52y
**III.5**	38	F	No	−/−	−/−	-	No	I	65	SR	-	
**III.6**	36	F	No	−/−	−/−	-	No	I	60	SR	-	
**III.7**	57	F	Yes	+/−	−/−	46	NA	I	NA	NA	NA	SCD 57y
**III.9**	40	M	Yes	NA	NA	NA	NA	NA	NA	NA	NA	SCD 40y
**III.11**	42	F	No	−/−	+/−	-	No	I	60	SR	-	
**III.13**	40	M	No	−/−	+/−	-	No	I	60	SR	NA	
**III.15**	44	F	No	−/−	+/−	-	No	I	60	SR	-	
**III.16**	39	F	No	−/−	+/−	-	No	I	63	SR	-	
**III.18**	53	F	Yes	+/−	−/−	38	Yes	III	33	SR	AVB-III°, LBBB	Prophylactic ICD 42y, CRT-D 52y
**IV.1**	22	M	No	−/−	−/−	-	No	I	60	SR	-	
**IV.2**	35	M	Yes	+/−	−/−	26	early DCM	I	50	SR	LPH	
**IV.3**	20	F	No	−/−	−/−	-	No	I	60	SR	-	
**IV.4**	19	F	No	+/−	−/−	-	early DCM	I	50	SR	-	
**IV. 5**	23	M	Yes	+/−	−/−	23	No	I	60	SR	AVB-I°	
**IV. 6**	23	F	No	+/−	−/−	-	No	I	55	SR	-	
**IV.7**	19	M	No	−/−	−/−	-	No	I	64	SR	-	
**IV.11**	27	F	No	+/−	−/−	-	No	I	55	SR	-	
**IV.12**	20	F	No	−/−	−/−	-	No	I	55	SR	-	

AF: atrial fibrillation; AVB: atrioventricular block; CRT-D: cardiac resynchronization therapy defibrillator; DCM: dilated cardiomyopathy; F: female; ICD: implantable cardioverter-defibrillator; LAH: left anterior hemiblock; LBBB: left bundle branch block; LPH: left posterior hemiblock; M: male; NA: not available; NYHA: New York Heart Association; SCD: sudden cardiac death; SR: sinus rhythm; PM: pacemaker; y: year.

## Supplementary Materials

The following supporting information can be downloaded at: https://www.mdpi.com/article/10.3390/ijms232012230/s1.
