# Supplementary material for: Genotype Complements the Phenotype: Identification of the Pathogenicity of an LMNA Splice Variant by Nanopore Long-Read Sequencing in a Large DCM Family
Source: Int J Mol Sci. 2022 Oct 13;23(20):12230. doi: 10.3390/ijms232012230 (PMC9602549; doi:10.3390/ijms232012230)

Supplemental Figure Legends

Supplemental Figure S1: Undetected variant by using Sanger sequencing and HaloPlex. The etiology of the disease using Sanger sequencing and previous versions of NGS (HaloPlex) had remained unclear for a long time because of allelic dropout **(A)** and low coverage **(B)**. P: primer.

Supplemental Figure S2: LMNA-iPSCs show typical pluripotent characteristics. **A:** LMNA-iPSCs show typical stem cell morphology and are positive for alkaline phosphatase activity (ALP) (scale bar= 200 µm). They show expression of the pluripotency-related proteins OCT4, NANOG, SOX2 and LIN28 visualized by immunofluorensence staining (scale bar= 100 µm). **B:** RT-PCR of pluripotency related genes. The iPSC from both LMNA patients show expression of *GDF3, NANOG, OCT4, SOX2, FOXD3,* and *LIN28*. Patient-fibroblasts (FB) serve as negative control and previously published iPSCs as a positive control (PC). **C:** In vitro differentiation potential of LMNA-iPSC. Culture in embryoid bodies induces spontaneous differentiation of iPSC showing cells from all three germ layers: alpha-feto protein (AFP; endoderm), alpha-smooth muscle actin (α-SMA), and beta-III-Tubulin (β-III-TUB).

Supplemental Figure S3: Ca^{2+} kinetics of LMNA-iPSC-CMs. Ca^{2+} cycling was assessed with Ca^{2+}-sensitive probe Fluo4. **A:** Values for time to peak are blotted for 4 cardiac differentiations of control (n = 70), 2 differentiations for LMNA 1 (n = 35) and 3 differentiations for LMNA 2 (n = 53). **B** and **C:** The LMNA-iPSC-CMs show a physiological reaction to Isoprenaline (Iso) shown by decreased Ca^{2+} time to peak and transient decay time values after Iso stimulation (100 nM). Numbers same as in A.

Supplemental Figure S1

A)

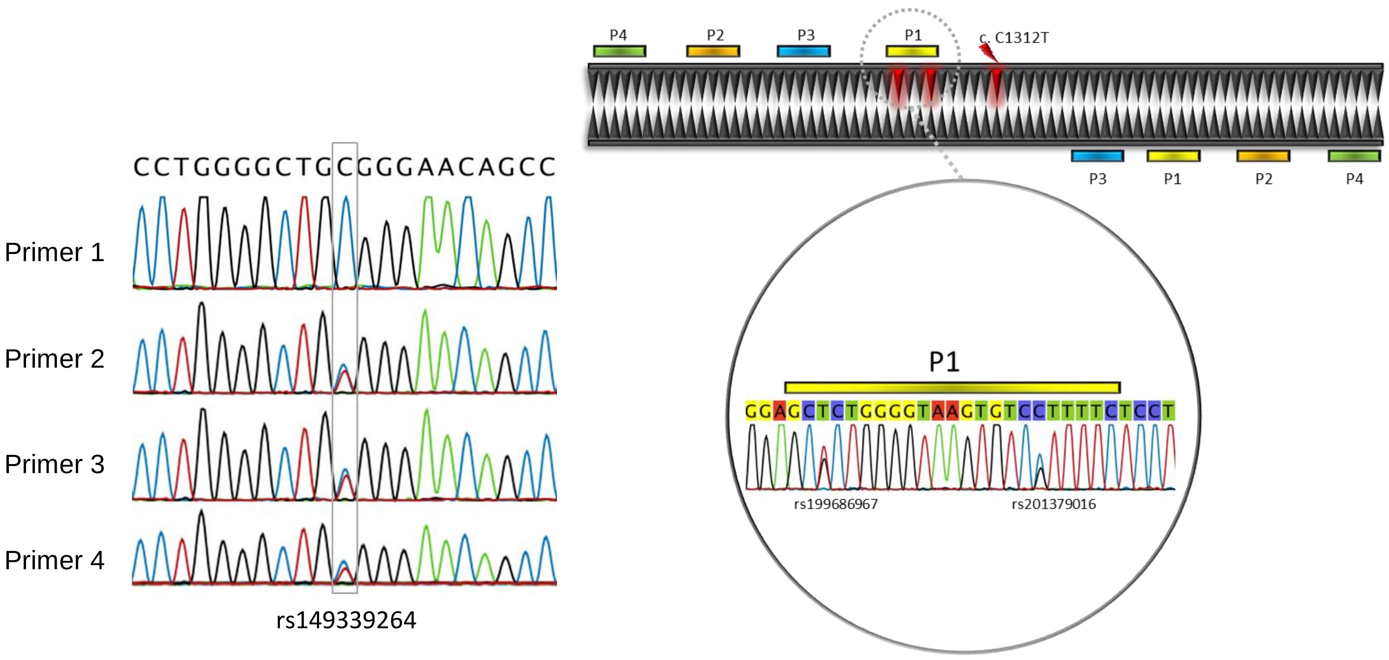

B)

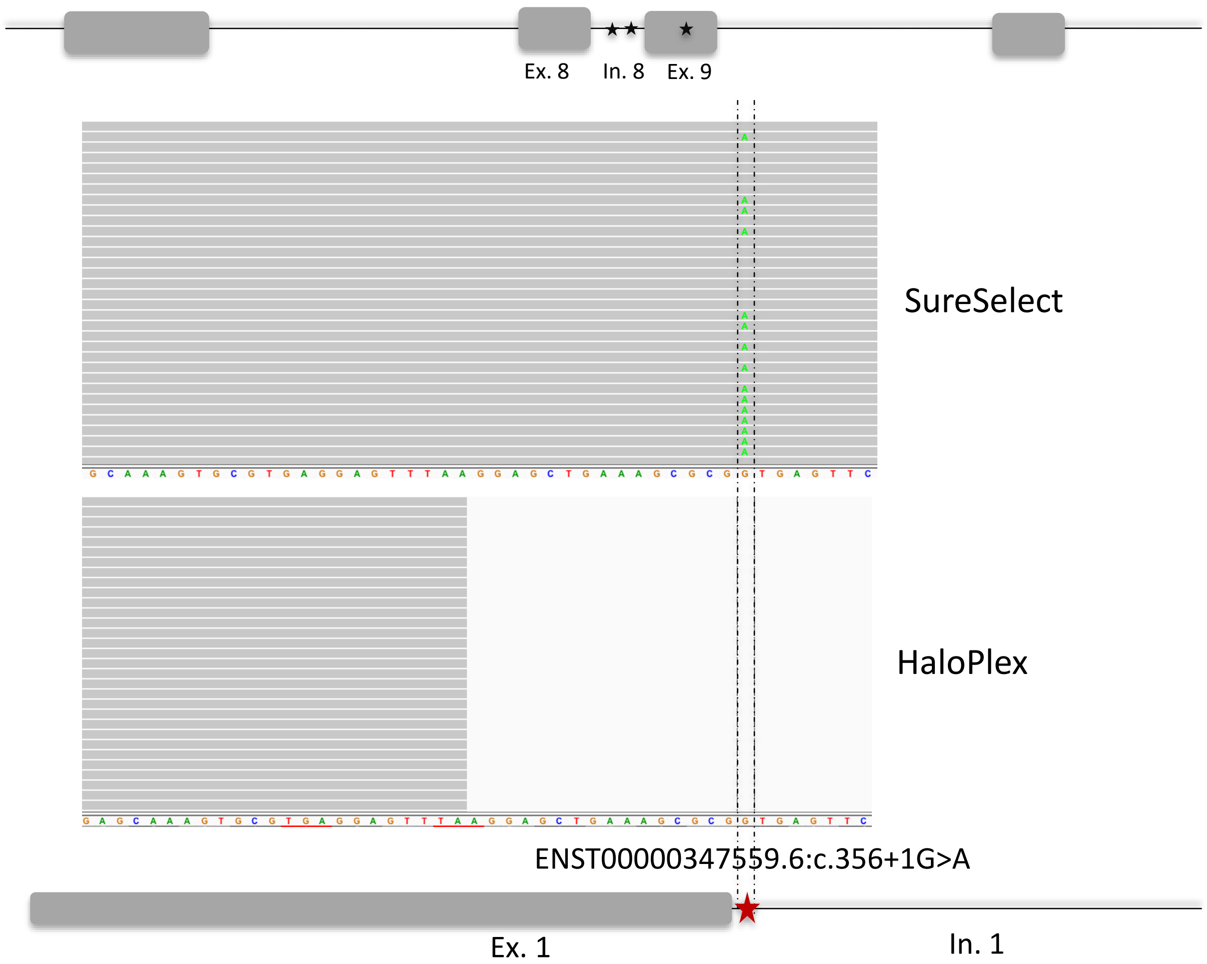

Supplemental Figure S2

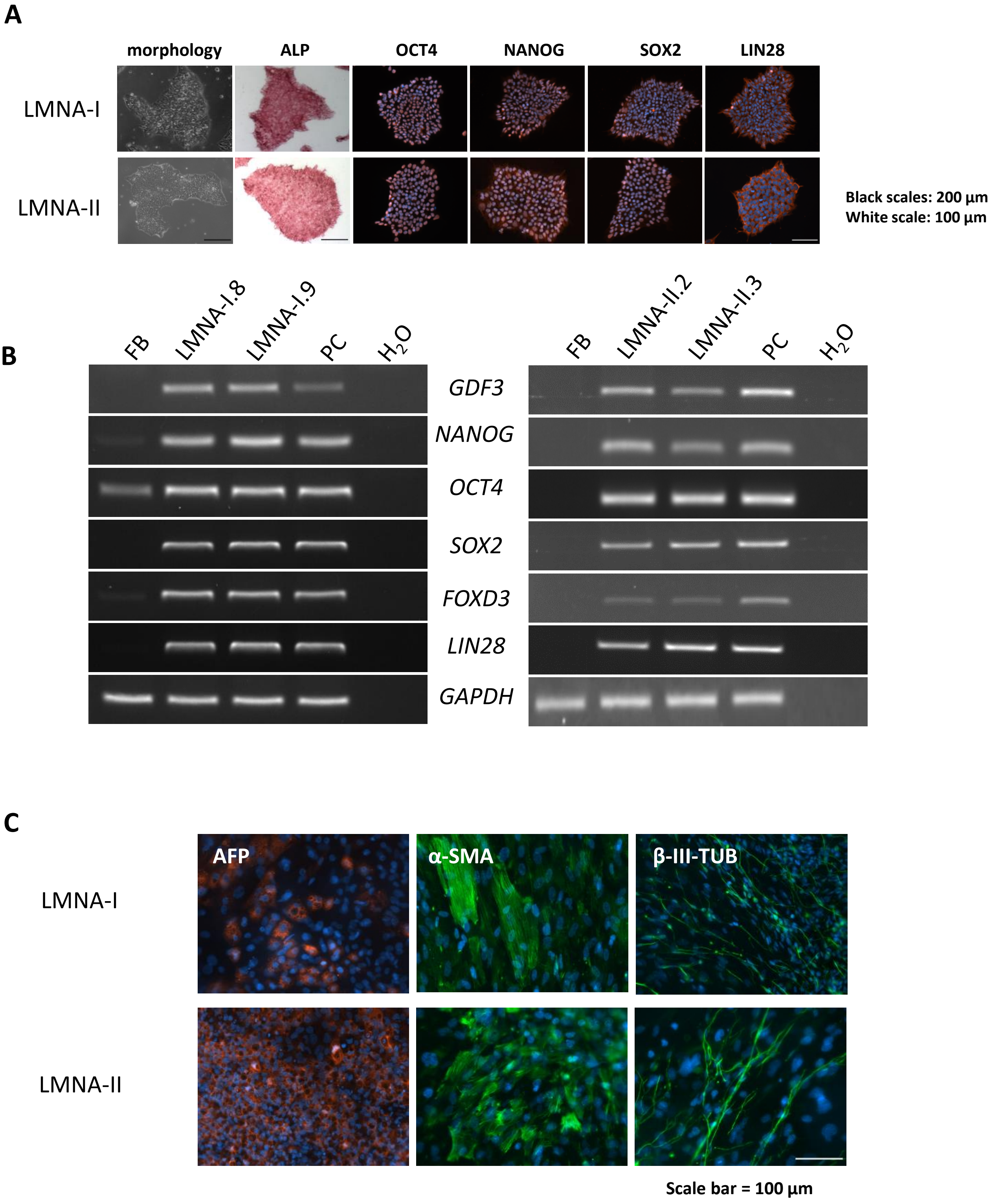

Supplemental Figure S3

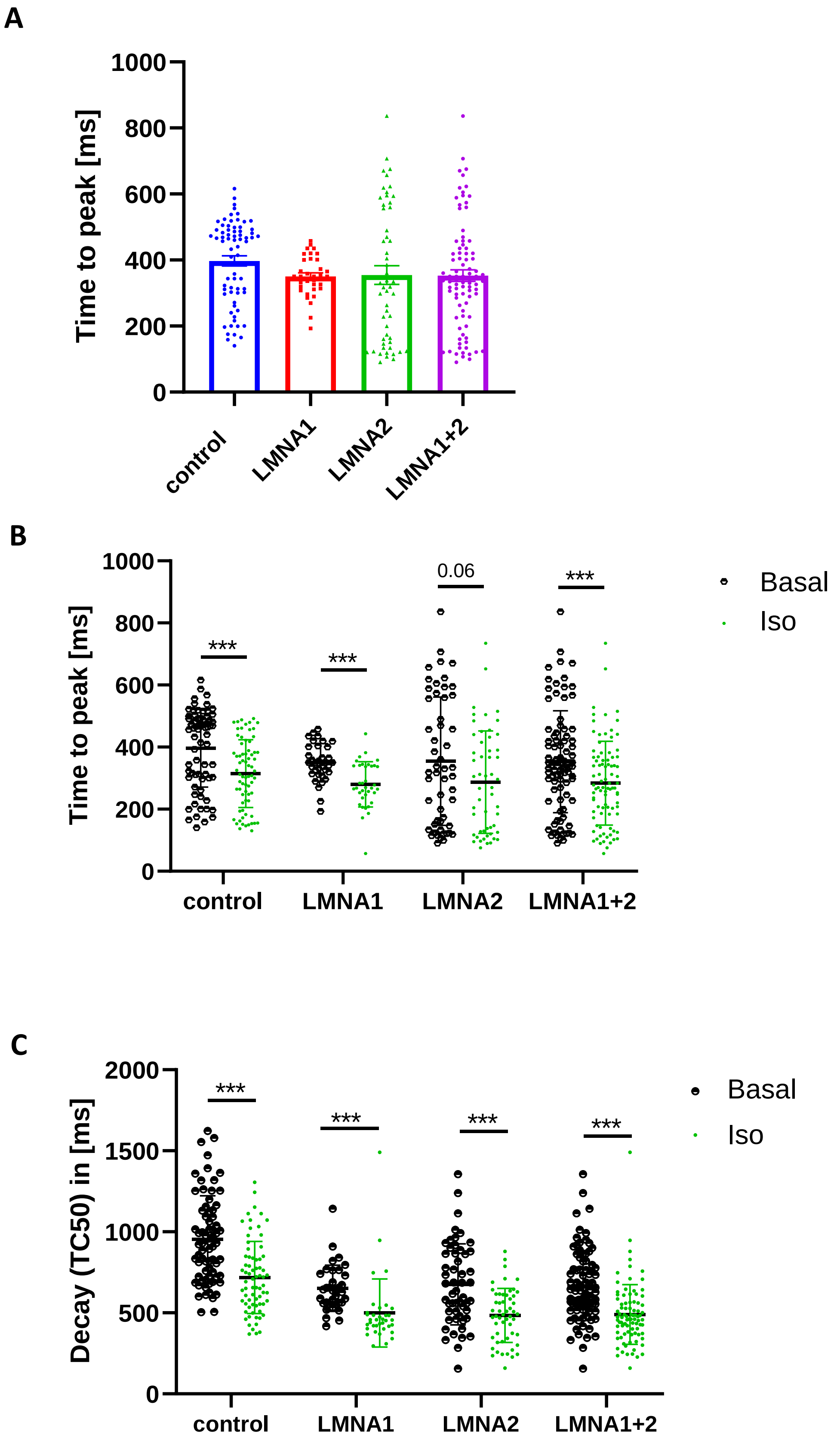

Supplement: Supplementary file 1 [file ijms-23-12230-s001.zip › ijms-1926188-supplementary.pdf]
